# Use of Potentially Inappropriate Medications for People with Diabetes as a Confounding Factor in Alzheimer's Diagnosis: An Analysis Using the ADNI Database

**DOI:** 10.1002/alz70857_105637

**Published:** 2025-12-25

**Authors:** Taiane Santos Garcia, Sheyla Velasques Paladini, João Pedro Ferrari‐Souza, Lucas Uglione Da Ros, Eduardo R. Zimmer, Agnes Nogueira Gossenheimer

**Affiliations:** ^1^ Universidade Federal do Rio Grande do Sul, Porto Alegre, Rio Grande do Sul, Brazil; ^2^ Universidade Federal do Rio Grande do Sul, Porto Alegre, RS, Brazil; ^3^ Universidade Federal Do Rio Grade Do Sul, Porto Alegre, Rio Grande do Sul, Brazil

## Abstract

**Background:**

The Alzheimer's Disease Neuroimaging Initiative (ADNI) aims to improve Alzheimer's disease (AD) clinical trials. Patients with diabetes often use potentially inappropriate medications (PIMs), which can impact cognitive function, influence neurodegenerative biomarkers, and confound Alzheimer's diagnosis. This study leverages data from the Alzheimer's Disease Neuroimaging Initiative (ADNI) to investigate whether the use of PIMs by individuals with diabetes is associated with cognitive changes or biomarkers that could be misinterpreted as Alzheimer's disease.

**Method:**

The study utilizes data from the Alzheimer's Disease Neuroimaging Initiative (ADNI), encompassing medication history, diabetes diagnosis, cognitive performance, and biomarkers such as PET/MRI imaging, beta‐amyloid, and tau levels. Potentially inappropriate medications (PIMs) were identified using the Beers criteria, focusing on medications that pose high risks for older adults with diabetes. The study population includes individuals with diabetes from the ADNI database, both with and without a diagnosis of Alzheimer's disease.

**Result:**

Total of 411 individuals, including 310 with diabetes, were enrolled in the study. The median age (IQR) was 74.7 years [50.1–90.0], with 56% being men and a median of 15.2 years of formal education. Among them, 96 individuals were identified as PIM users. The PIMs found were sulfonylureas (all, including short‐ and longer‐acting) and short‐ or rapid‐acting insulin. These medications can cause hypoglycemia, which in the elderly often manifests as mental confusion, agitation, mood and cognitive changes. Further analyses will investigate the association between PIM use and cognitive decline or biomarker alterations in this population.

**Conclusion:**

Findings may indicate that PIMs, widely used by older adults with diabetes, have the potential to influence cognitive outcomes and Alzheimer's biomarkers, emphasizing the need for rational prescribing in vulnerable populations. This study contributes to improving differential diagnosis and promoting safer pharmacotherapeutic practices in older adults with diabetes.

